# Endovascular repair of type A aortic intramural hematoma accompanied by aberrant right subclavian artery and Kommerell’s diverticulum: a case report

**DOI:** 10.1186/s12872-020-01504-2

**Published:** 2020-05-13

**Authors:** Sizheng Xiong, Daoquan Wang, Jun Li

**Affiliations:** Division of Cardiothoracic and vascular Surgery, Tongji Hospital, Tongji Medical College, Huazhong University of Science and Technology, Wuhan, 430000 China

**Keywords:** Aortic intramural hematoma, Kommerell’s diverticulum, Aberrant right subclavian artery

## Abstract

**Background:**

The lesions of aberrant right subclavian artery, Kommerell’s diverticulum and type A aortic intramural hematoma are rare, and we usually treat them with open surgery. In some cases patients have increased risk to undergo surgery, the experiences of endovascular or medical treatment are limited.

**Case presentation:**

Here we reported a case of a 53-year-old man with these three entities present with chest and back ache and attempted a novel approach, thoracic endovascular aortic repair, in the absence of surgical treatment. The patient lived over 5 years and this case provides initial experience and lesson about the endovascular and medical management of the uncommon and dangerous disease- type A aortic intramural hematoma with aortic congenital malformation.

**Conclusion:**

Thoracic endovascular aortic repair with medical treatment may be a potential alternative approach for type A aortic intramural hematoma.

## Background

**Fig. 1:**
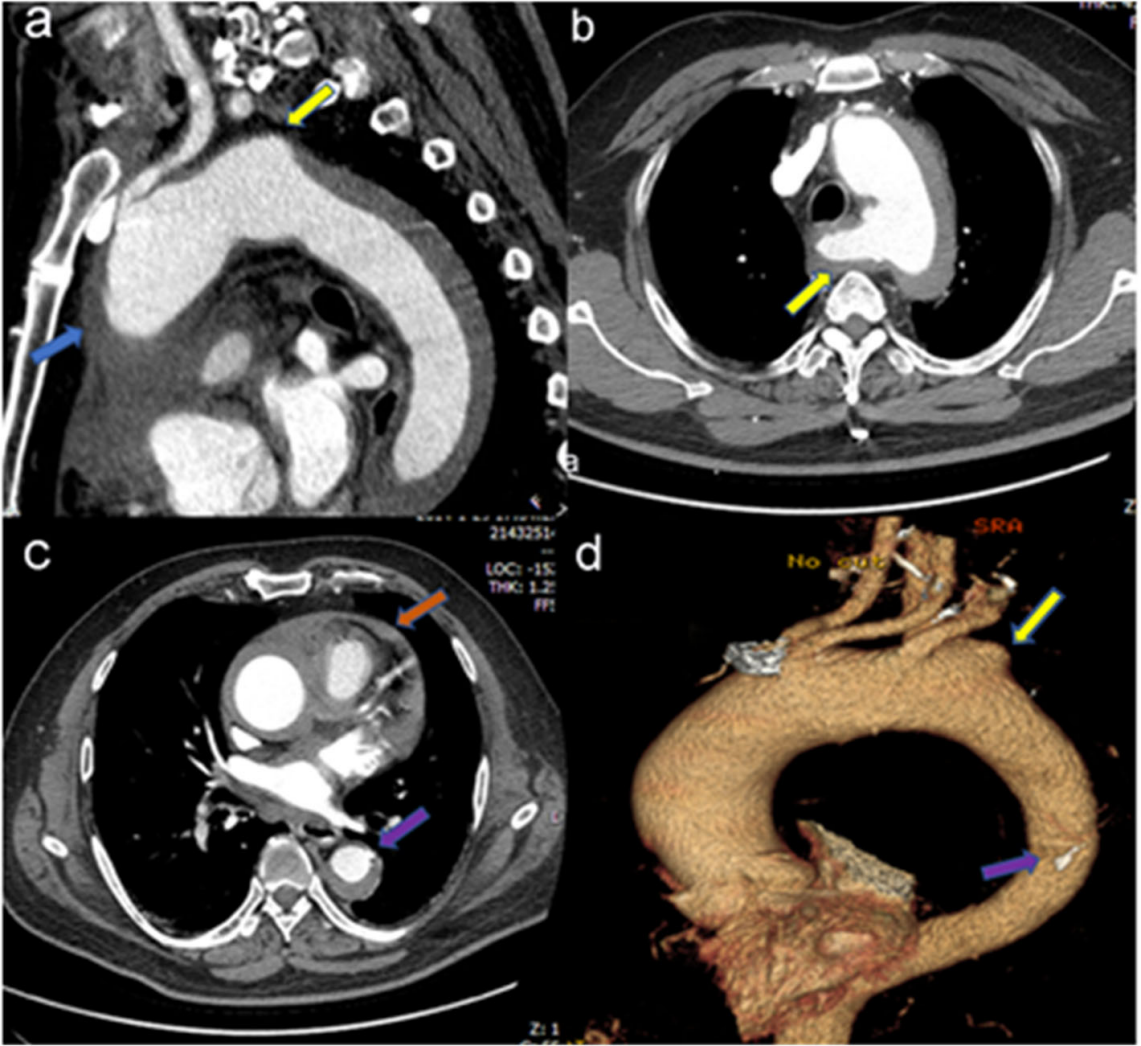
The computed tomography angiography of the patient on admission. **a** The ascending aortic intramural hematoma (blue arrow) and the Kommerell’s diverticulum (KD, yellow arrow). **b** KD, (yellow arrow): the aneurysmal aortic dilatation occurs at the origin of the aberrant right subclavian artery (ARSA). **c** A penetrating atherosclerotic ulcer (PAU, purple arrow) located in descending aorta and pericardial effusion (brown arrow). **d** PAU (purple arrow) and KD (yellow arrow) at three-dimensions reconstruction

Aortic intramural hematoma (IMH) is a clinical entity characterized by hemorrhage within the media of aortic wall in absence of an intimal disruption, generally caused by rupture of the vasa-vasorum or intimal fracture induced by the progress of the atherosclerotic plaque, and Stanford type A IMH (TAIMH) is defined as the lesion involving the ascending aorta (Fig. 1a) [1]. Aberrant right subclavian artery (ARSA) is a congenital anomaly of the aortic arch, originating directly from the aorta after the left subclavian artery and occurring in 0.5 to 1% of the population [2]. Rarely, an aneurysmal aortic dilatation occurs at the origin of the ARSA and is termed as Kommerell’s diverticulum (KD, Fig. 1b, d) [2]. Surgery is regarded as the most effective way to treat KD or TAIMH at present, however, a novel approach was tried in our center because the patient refused the surgical treatment.

## Case presentation

**Fig. 2:**
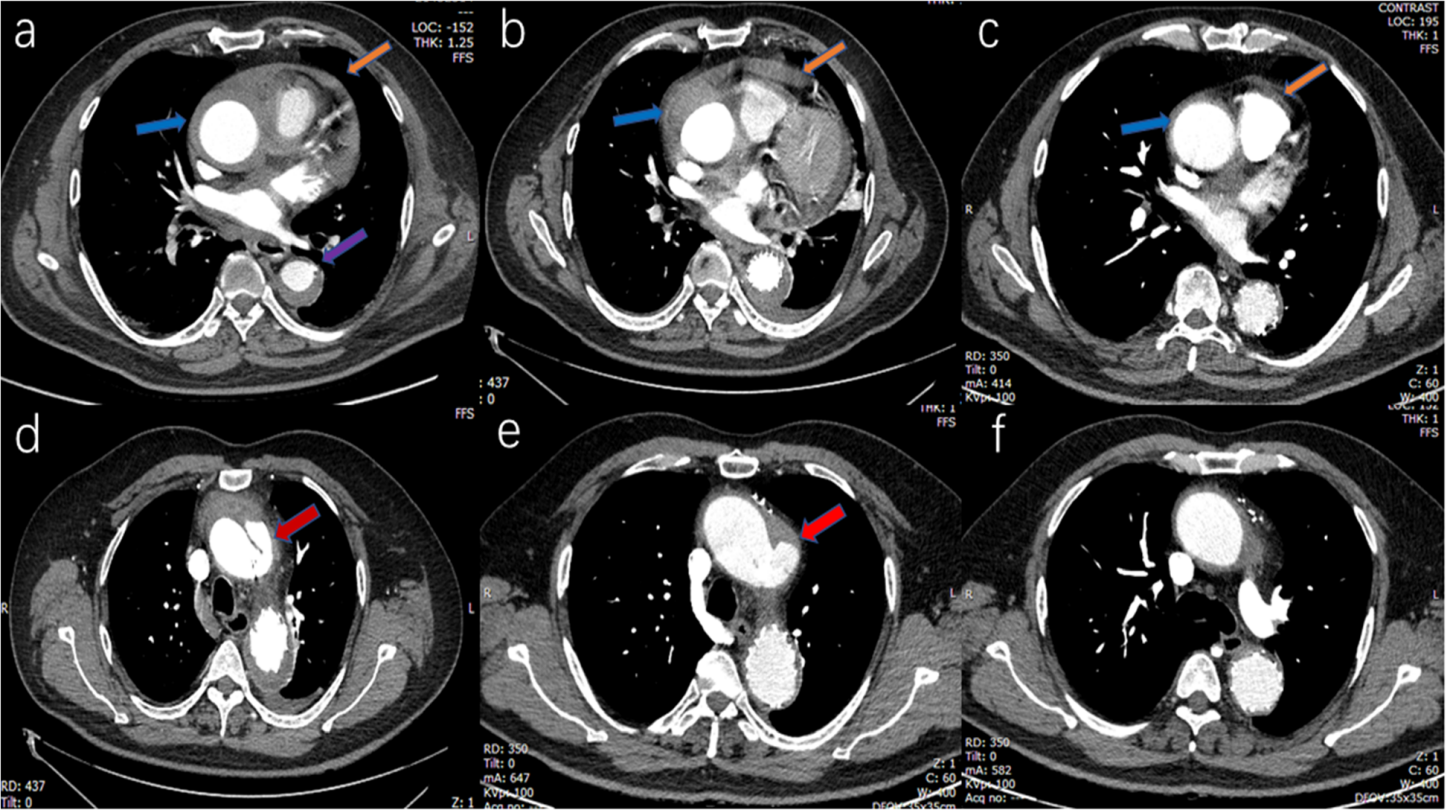
The changes on computed tomography angiography between admission and after thoracic endovascular aortic repair (TEVAR). **a** On admission: a penetrating atherosclerotic ulcer (PAU, purple arrow) located in descending aorta, the ascending aortic intramural hematoma (blue arrow) and pericardial effusion (brown arrow). **b** 2 weeks after TEVAR: PAU was excluded but the maximal ascending aortic diameter (MAAD) and the maximal ascending aortic hematoma thickness (MAHT, blue arrow) increased, and the pericardial effusion (brown arrow) and pleural effusion progressed. **c** 3 months after TEVAR: the MAAD decreased with the MAHT (blue arrow), the pericardial effusion (brown arrow) and the pleural effusion nearly disappearing. **d** 2 weeks after TEVAR: an aortic arch dissection (red arrow) distal to ascending aorta occurred. **e** 3 months after TEVAR: the aortic arch dissection (red arrow) stabilized. **f** 3 months after TEVAR: a dissection was confined within the aortic arch

A 53-year-old man with the comorbidities of hypertension and chronic renal insufficiency was referred to our center with the complaint of chest and back ache for 1 h. Electrocardiogram suggested the acute anteroseptal myocardial infarction in emergency room, then, coronary angiography was applied and found no coronary stenosis. Finally, he was diagnosed with ARSA, KD and TAIMH with the presence of pericardial effusion and a penetrating atherosclerotic ulcer (PAU, Fig. 1c, d) located in descending aorta by computed tomography angiogram (CTA). Meantime, there was a vascular ring including left aortic arch, right aberrant subclavian artery and right ligamentum. The patient’s condition was stabilized by medical. Because of TAIMH and the pericardial effusion it induced, we recommended surgery firstly, but he refused surgery for the high risk of traditional vascular replacement with his worsening renal function. On the basis of the assumption that the TAIMH was induced by the PAU in this case and could be treated by excluding the PAU, thoracic endovascular aortic repair (TEVAR) was performed 5 days after admission with the consent of the patient. We covered the PAU located in descending aorta and preserved the ARSA to reduce the risk of right arm malperfusion and stroke. The procedure was under general anesthesia and the blood pressure and heart rate were kept steady in this period. We implanted the stent via femoral incision and the proximal landing zone of the graft was just distal to the ostium of the ARSA. The stent we used was Relay (Bolton Medical, Sunrise, FL, USA) with the size of 36 mm*150 mm and oversize of 10%. The patient continued receiving medication to lower systolic blood pressure ≤ 130 mmHg and heart rate ≤ 70 beats/min after TEVAR and no complication occurred, but it showed that the maximal ascending aortic diameter (MAAD) and the maximal ascending aortic hematoma thickness (MAHT) increased, from 59.1 mm to 62.3 mm and 9.3 mm to 17.4 mm respectively, meanwhile, the pericardial effusion and pleural effusion progressed and a localized aortic arch dissection occurred at the first CTA review 2 weeks after TEVAR (Fig. 2a, b, d). Although further treatment should be offered, the patient was only willing to undertake medical treatment and refused the surgery or reintervention. After discharge, the patient was prescribed anti-hypertensive drugs and examined by CTA 3 months after TEVAR and it showed that the MAAD decreased to 50.1 mm with the MAHT; the pericardial effusion and the pleural effusion nearly disappeared and the aortic arch dissection stabilized (Fig. 2c, e, f). At the last follow up 5.5 years after TEVAR, the patient was alive without progression of the disease.

## Discussion

Although the treatment of TAIMH were under consensus in different regions, surgery was advocated when TAIMH was accompanied by the PAU, which was a predictor of progress of the TAIMH [3]. However, Lyons et al. summarized endovascular management of TAIMH With a distal primary entry tear and there were no in-hospital mortality and 2 died in the short-term follow-up of 24 months in the 17 patients [4]. As for KD, aggressive treatment was proposed given the risk of rupture, but the operative mortality associated was high (16%) and the experience of endovascular exclusion was limited [5]. With the development of imaging technologies, the small intimal tear or rupture of atherosclerotic plaque which was found in the ward and some of them were confirmed during the operation within some patients with IMH, which was different from the classical definition or ‘aortic dissection without intimal tear’ [6–8]. Furthermore, Grimm et al. suggested the TAIMH could be induced by the atherosclerotic plaque rupture located in descending aorta, which was similar to our case [9]. Therefore, based on the previous studies, we assumed that the patient could benefit from the excluded PAU by TEVAR along with the basic medical therapy and took the strategy of ‘wait and see’ for the KD, which actually slowed down the progression of aneurysms by controlling the blood pressure and heart rates. But he was thought to experience progress of the disease not too long after the intervention for the reason that no complications were observed at the end of the intervention, which might be also relevant with the unclear causes of the IMH or veiled tear which was undetected within the imaging technology at present besides the lesion secondary to TEVAR [4]. The complexity of these entities should not be neglected whether treatment strategy was carried out. ARSA would increase the difficulty for total arch replacement and TAIMH with KD had a negtive impact on landing and release of the stent as well. For patients who had comorbidities and higher risks of surgery, TEVAR had less invasive incision and complications but required serious follow-up additionally. Although the patient survived over 5 years, the problem and challenge emerging after TEVAR suggested that it should be prudent to treat TAIMH accompanied with PAU by TEVAR, especially with other vascular malformations existing.

TEVAR with medical treatment may be a potential alternative approach for TAIMH accompanied by PAU located in descending aorta when surgery is unavailable and the researches of larger sample size and longer follow-up are required to verify the rationality of this strategy.

## Data Availability

All available information is contained within the present manuscript.

## Abbreviations

IMH: Aortic intramural hematoma
TAIMH: Stanford type A aortic intramural hematoma
ARSA: Aberrant right subclavian artery
KD: Kommerell’s diverticulum
PAU: Penetrating atherosclerotic ulcer
TEVAR: Thoracic endovascular aortic repair
CTA: Computed tomography angiogram
MAAD: Maximal ascending aortic diameter
MAHT: Maximal ascending aortic hematoma thickness
